# Supplementary material for: MetaRibo-Seq measures translation in microbiomes
Source: Nat Commun. 2020 Jun 29;11:3268. doi: 10.1038/s41467-020-17081-z (PMC7324362; doi:10.1038/s41467-020-17081-z)
Supplement: Supplementary file 10 — Supplementary Data 7 [file 41467_2020_17081_MOESM10_ESM.zip › File2/Confidence_VeryHigh_Taxonomy/108843_out.krona.html]

Javascript must be enabled to view this page.

members
magnitude
magnitudeUnassigned
count
unassigned
taxon
rank

108843\_out

5

2
5
superkingdom

5
976
phylum

200643
5
class

order
5
171549

171552
4
family

4
838
genus

2

SRS049959\_contig\_number\_32026SRS077849\_contig\_number\_29015
2292054
species

species

SRS012969\_contig\_number\_1574SRS023526\_contig\_number\_contig-100\_2072.222919
2
2292365

family
1
1853231

genus
1
283168

28118

SRS012849\_contig\_number\_23485
1
species
